# Local Positioning System-Derived External Load of Female and Male Varsity Ice Hockey Players During Regular Season Games

**DOI:** 10.3389/fphys.2022.831723

**Published:** 2022-02-25

**Authors:** Alexander S. D. Gamble, Jessica L. Bigg, Danielle L. E. Nyman, Lawrence L. Spriet

**Affiliations:** ^1^Department of Human Health and Nutritional Sciences, University of Guelph, Guelph, ON, Canada; ^2^Human Performance and Health Research Laboratory, University of Guelph, Guelph, ON, Canada; ^3^School of Kinesiology and Health Studies, Queen’s University, Kingston, ON, Canada

**Keywords:** player tracking, wearable technology, athlete monitoring, individualized speed thresholds, team sport

## Abstract

**Purpose:**

The purposes of this study were to quantify the external load for female and male varsity ice hockey players during regular season games using a local positioning system (LPS), compare LPS-derived external load between sexes and positions, and compare skating distances in absolute and relative speed zones.

**Methods:**

Data were collected for 21 female (7 defense, 14 forwards; 20.0 ± 1.4 yrs., 69.1 ± 6.7 kg, 167.1 ± 5.4 cm) and 25 male (8 defense, 17 forwards; 21.9 ± 1.1 yrs., 85.9 ± 5.4 kg, 181.1 ± 5.2 cm) varsity ice hockey players. Measures included skating distance (total, and in absolute and relative speed zones), peak skating speed, peak acceleration and deceleration, accumulative acceleration load, and number of accelerations, decelerations, turns, skating transitions, direction changes, and impacts.

**Results:**

Female and male players had a high external load during games, with average peak skating speeds >28 km/h and average skating distances >4.4 km. Most LPS-derived measures showed greater external load in males than females (*p* < 0.05). Forwards skated further at higher speeds compared to defense in both sexes (*p* < 0.001). Skating distances were significantly different when comparing absolute and relative speed zones (*p* < 0.001), with absolute speed zones potentially overestimating skating at very slow, very fast, and sprint speeds and underestimating skating at slow and moderate speeds.

**Conclusion:**

This was the first study to measure external load in female ice hockey players with a LPS. Both female and male varsity players had high external loads during games, with forwards having greater external load at higher intensities and defense having greater external load at lower intensities. Sex and positional differences outline the importance of individualized athlete monitoring.

## Introduction

Athlete load can be separated into internal and external load, with the external load of an athlete defined as the amount of work objectively quantified during training or competition (Bourdon et al., 2017). Traditionally, athlete external load was primarily measured by time or distance of exposure. Time–motion and video analysis have been used to investigate movement characteristics and skating in female and male varsity ice hockey players (Jackson et al., 2016, 2017). This research has provided important introductory analyses of ice hockey movements, but quantifying movements in more detail using video analysis is difficult given that it can be time-consuming and subjective (Dobson and Keogh, 2007; Dellaserra et al., 2014). Recently, local positioning systems (LPS) have allowed for quantification of external load and athlete movement tracking in indoor sports where global positioning systems (GPS) are not feasible (Brooks et al., 2020; Douglas and Kennedy, 2020; Vigh-Larsen et al., 2020; Gamble et al., 2021a,b; Vázquez-Guerrero and Garcia, 2021).

Discussion of the available and most appropriate measures used to quantify external load in team sports is ongoing as technology continues to improve (Ortega et al., 2021). While timing gates, video analysis, and other wearable technology have all been used to measure external load in ice hockey (Van Iterson et al., 2017; Lignell et al., 2018; Link et al., 2019; Stetter et al., 2019; Douglas et al., 2019a; Allard et al., 2020), they do not provide the same amount of information as LPS. Based on positional information in indoor sports (similar to outdoor GPS position-tracking), distance travelled, speed, acceleration, and deceleration can all be determined. Furthermore, GPS, video *x* and *y* coordinate position, and an infrared camera system have been used to validate LPS in several instances (Bastida Castillo et al., 2018, 2019a,b; Hoppe et al., 2018; Luteberget et al., 2018; Blauberger et al., 2021).

Until recently, accelerometry has been the primary measure of external load in female ice hockey players, which has been applied to comparisons of match outcome, training and competition, and elite and sub-elite players (Douglas et al., 2019a,b, 2020). However, there have been two recent studies that measured external load in ice hockey games using LPS, and both studies were in males (Douglas and Kennedy, 2020; Vigh-Larsen et al., 2020). Given the differences in female and male anthropometrics, physiology, and competition rules (Hunter, 2014), studies using LPS to measure external load are needed to provide this information for female ice hockey players, especially during games.

Previous research reported faster skating speeds or higher intensity external load in forwards compared to defense in both female and male players using various methods of athlete monitoring (Lignell et al., 2018; Douglas et al., 2019a; Allard et al., 2020; Douglas and Kennedy, 2020). While athlete load research is relatively new in ice hockey and more studies are needed to determine the most appropriate LPS metric to measure external load, rugby research has suggested the importance of considering relative measures of external load to prevent misrepresentation of individual workloads between positions or sexes (Clarke et al., 2015; Reardon et al., 2015; Takamori et al., 2020; Nyman and Spriet, 2021). For example, distance travelled in what are considered physiologically demanding or high-intensity speed zones could change drastically between absolute thresholds previously outlined in ice hockey and those that consider each player’s peak speed (Lignell et al., 2018). The differences within the game of ice hockey between sexes and positions outline the need for this consideration, especially if these measures are being used for a method of monitoring workload.

Therefore, the purposes of this study were to: (i) quantify the external load for both female and male varsity ice hockey players during regular season games using LPS, (ii) compare LPS-derived external load between sexes and positions, and (iii) compare skating distance in absolute and relative speed zones as a measure of external load. It was hypothesized that LPS-derived external load measures would differ between sexes, and forward players would have faster skating speeds and higher intensity of external load when compared to defense.

## Materials and Methods

### Participants

Twenty-one female (7 defense, 14 forwards) and 25 male (8 defense, 17 forwards) varsity ice hockey players participated in this research during the 2019–2020 season. The mean (± SD) age, weight, and height of the subjects were 20.0 ± 1.4 yrs., 69.1 ± 6.7 kg, 167.1 ± 5.4 cm for females and 21.9 ± 1.1 yrs., 85.9 ± 5.4 kg, 181.1 ± 5.2 cm for males. Players were informed of all protocols, requirements, and risks, both verbally and in writing, prior to obtaining oral and written consent. All procedures were approved by the Research Ethics Board of the University and conformed to the Declaration of Helsinki and meet the ethical standards of the journal.

### Study Design

External load data were collected for men’s (13 games) and women’s (11 games) varsity ice hockey teams during the 2019–2020 regular season using an ultra-wideband LPS (Kinexon, Munich, Germany). The reliability and validity of this technology has recently been reported for ice hockey (Gamble et al., 2021b). Eighteen skaters (6 defense, 12 forwards) were selected to participate in each game by respective coaches. All games were standardized by league regulation and played on the same Olympic-size ice surface. Data collection only occurred during gameplay, which did not include warmups or intermissions.

#### LPS-Derived External Load

**Figure 1:**
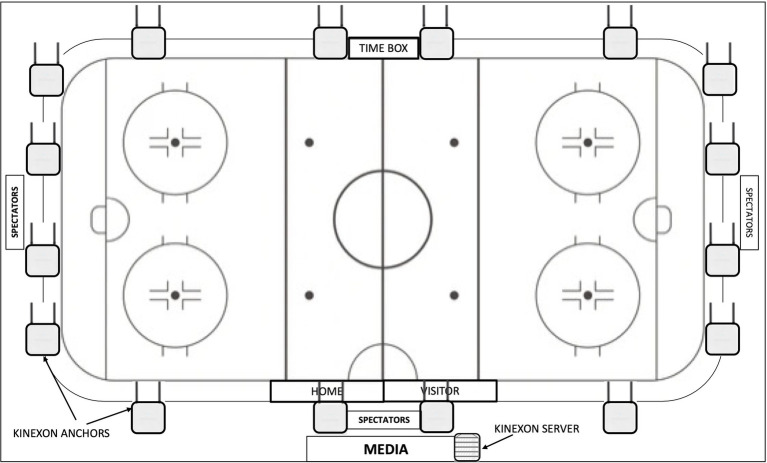
Schematic diagram outlining the location of one server and 16 anchors that allow for on-ice player tracking during games and practices using an ultra-wideband local positioning system (Kinexon, Munich, Germany).

The LPS incorporated specific local network access, one Power over Ethernet switch and one server (located in the media booth), 16 anchors (secured to the rafters), and one sensor for each participant (secured in a patch on the posterior side of the shoulder pads near the superior 1/3 of the scapula; Figure 1). Communication between anchors and the player sensors (though ultra-wideband channels ranging from 3244.88–4742.40 MHz) allowed for collection of real-time data, which was then transmitted to the server *via* hardwired connection. Using the local network, the LPS platform and data (sampling rate of 20 Hz) could be retrieved on a secure computer or tablet.

Definitions of external load LPS metrics were adapted from One and Media-KNX ONE Hockey Metrics (Kinexon, Munich, Germany). Recorded metrics included: (i) skating distance: distance covered based on changes in on-ice position (total and separated into specific speed zones), (ii) peak skating speed: maximum instantaneous speed based on changes in on-ice position and time, (iii) peak acceleration: maximum instantaneous acceleration based on changes in on-ice speed and time, (iv) peak deceleration: maximum instantaneous deceleration based on changes in on-ice speed and time, (v) number of accelerations and decelerations: frequency of player accelerations and decelerations >|2 m/s^2^|, (vi) sharp (turn radius <2 m) and wide (turn radius >2 m) turns, (vii) skating transitions: number of transitions from forward-to-backward or backward-to-forward skating, (viii) number of changes of direction: frequency of changes in on-ice motion direction following a deceleration and prior to an acceleration, and (ix) number of impacts: frequency of collisions (triggered by a G force >3 g on the sensor) with the ice, boards, or another player.

#### Determining Skating Distance in Speed Zones

Absolute and relative speed zones were used to provide a measure of intensity to the skating distance travelled (Table 1). Female and male absolute speed zones used in the present study were based on those previously outlined in elite male ice hockey literature (Lignell et al., 2018). Relative speed zones were determined by first identifying the peak game speed during the season for each player and then calculating percentages of the peak speed accordingly, as previously done by other team sport research (Reardon et al., 2015; Takamori et al., 2020; Nyman and Spriet, 2021; Table 1).

**Table 1:** Absolute and relative speed zones used to examine intensity of skating distances.

	Absolute speed zones		Relative speed zones	
	Speed (km/h)	% Peak speed	Speed for females (km/h)	Speed for males (km/h)
Very slow	1.0–10.9	<20%	<6.0	<6.6
Slow	11.0–13.9	20–39%	6.1–12.0	6.7–13.2
Moderate	14.0–16.9	40–59%	12.1–18.1	13.3–19.7
Fast	17.0–20.9	60–79%	18.2–24.1	19.8–26.4
Very fast	21.0–24.0	80–90%	24.2–27.1	26.5–29.7
Sprint	>24.0	>90%	>27.1	>29.7

### Statistical Analysis

All data are presented as means ± SD and were analyzed using STATA/IC 15.0 software (College Station, TX) and SPSS version 26 (Armonk, NY). Q–Q plots were used to visually confirm normality. Due to the differences in female and male ice hockey making it inappropriate for some of these groups to be combined for comparison, independent sample t-tests were used to compare means of LPS measures between females and males, and defense and forwards. Dependent sample *t*-tests were used to compare means of skating distance in absolute and relative speed zones. Statistical significance was accepted at *p* < 0.05.

## Results

### Peak Skating Speed and Skating Distance

Average peak skating speeds for regular season games were significantly greater for males in all players (31.16 ± 1.64 vs. 28.73 ± 1.51 km/h), defense (30.15 ± 1.68 vs. 28.52 ± 1.74 km/h), and forwards (31.73 ± 1.33 vs. 28.82 ± 1.39 km/h) compared to females (all *p* < 0.001). Forwards had greater peak speeds than defense in males (*p* < 0.001), but not in females (*p* = 0.197).

Average total skating distances were also significantly greater for males than females in all players (5381 ± 1705 vs. 4,489 ± 1,152 m), defense (5617 ± 1,441 vs. 5,154 ± 1,144 m; *p* = 0.042), and forwards (5250 ± 1828 vs. 4206 ± 1037 m). Defense skated further than forwards in females (*p* < 0.001), but not in males (*p* = 0.115).

**Figure 2:**
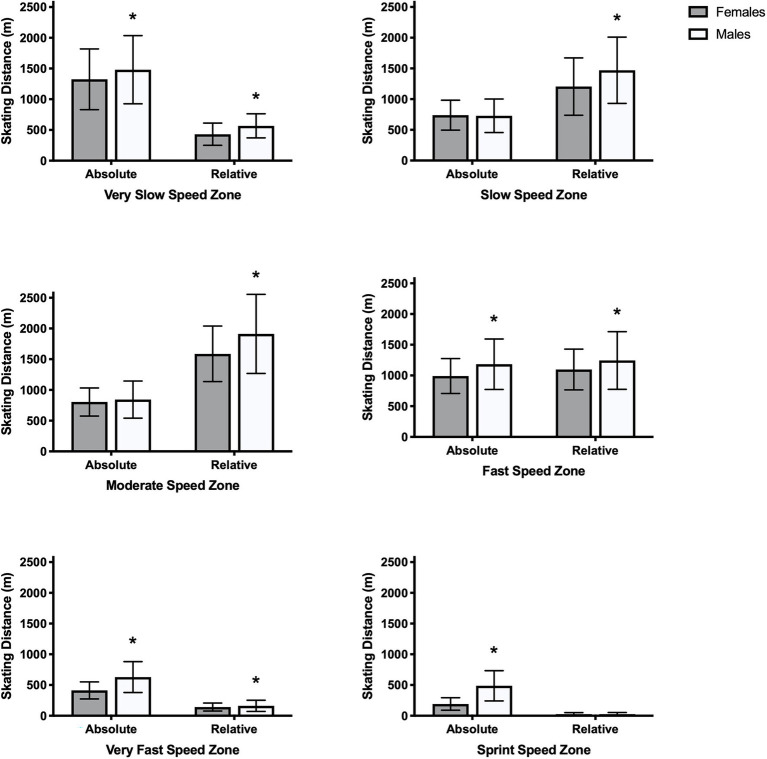
Mean skating distance travelled in very slow (absolute: 1.0–10.9 km/h; relative: <20%), slow (absolute: 11.0–13.9 km/h; relative: 20–39%), moderate (absolute:14.0–16.9 km/h; relative: 40–59%), fast (17.0–20.9 km/h; relative: 60–79%), very fast (21.0–24.0; relative: 80–90%), and sprint (absolute: >24.0 km/h; relative: >90%) speed zones for all female and male ice hockey players. Data are presented as mean ± SD. Absolute and relative skating distances were significantly different within sex for all speed zones (*p* < 0.05). ^*^represents a significant difference between skating distances for all females and males within absolute and relative speed zones (*p* < 0.05).

Skating distances in absolute speed zones were significantly greater for males than females in very slow, fast, very fast, and sprint speed zones (*p* < 0.004; Figure 2). Male forwards also skated further in very slow, fast, very fast, and sprint speed zones, while male defense skated further in the fast, very fast, and sprint speed zones (Table 2). Female and male defense skated significantly further than forwards in the very slow, slow, and moderate speed zones (*p* < 0.001). However, female and male forwards skated significantly further than defense in very fast and sprint speed zones (p < 0.001). There were no positional differences in the fast speed zone (*p* > 0.150). All skating distances were significantly different when comparing absolute and relative speed zones within sex and for all players (*p* < 0.001; Figure 2).

**Table 2:** Comparison of local positioning system measured absolute skating distances between females and males during regular season ice hockey games.

	Females	Males	Value of *p*
**Skating distance in very slow speed zone (1.0–10.9 km/h)—(m)**			
Defense	1790 ± 406	1837 ± 485	0.546
Forwards	1128 ± 384^*^	1,281 ± 490^*^	0.004
**Skating distance in slow speed zone (11.0–13.9 km/h)—(m)**			
Defense	936 ± 219	869 ± 245	0.093
Forwards	655 ± 203^*^	650 ± 256^*^	0.858
**Skating distance in moderate speed zone (14.0–16.9 km/h)—(m)**			
Defense	934 ± 225	935 ± 258	0.987
Forwards	748 ± 209^*^	791 ± 313^*^	0.175
**Skating distance in fast speed zone (17.0–20.9 km/h)—(m)**			
Defense	966 ± 274	1,131 ± 319	0.002
Forwards	1003 ± 288	1,212 ± 452	<0.001
**Skating distance in very fast speed zone (21.0–24.0 km/h)—(m)**			
Defense	337 ± 125	502 ± 194	<0.001
Forwards	445 ± 133^*^	699 ± 253^*^	<0.001
**Skating distance in sprint speed zone (>24.0 km/h)—(m)**			
Defense	158 ± 112	316 ± 176	<0.001
Forwards	207 ± 94^*^	584 ± 227^*^	<0.001

*Data are presented as means ± SD. Values of *p* represent comparisons of means between males and females within the same position*.

* *Represents a significant difference between defense and forwards (*p* < 0.05)*.

When considering relative speed zones, skating distances were significantly greater for males than females at all speeds, except the sprint speed zone (*p* < 0.010; Figure 2). Male defense and forwards also skated further at all speeds, except the sprint speed zone, when compared to their female counterparts (Table 3). Female and male defense skated significantly further than forwards in very slow and slow speed zones (*p* < 0.001), while only female defense skated further than forwards in the moderate speed zone (*p* < 0.001). Additionally, female and male forwards skated further than defense in fast and very fast speed zones (*p* < 0.003), and only female forwards skated further than defense in moderate and sprint speed zones (*p* < 0.007).

**Table 3:** Comparison of local positioning system measured relative skating distances between females and males during regular season ice hockey games.

	Females	Males	Value of *p*
**Skating distance in very slow speed zone (<20% peak speed)—(m)**			
Defense	597 ± 173	666 ± 175	0.021
Forwards	361 ± 130^*^	512 ± 187^*^	<0.001
**Skating distance in slow speed zone (20–39% peak speed)—(m)**			
Defense	1589 ± 418	1734 ± 474	0.061
Forwards	1042 ± 384^*^	1323 ± 517^*^	<0.001
**Skating distance in moderate speed zone (40–59% peak speed)—(m)**			
Defense	1853 ± 450	1965 ± 524	0.182
Forwards	1476 ± 404^*^	1882 ± 702	<0.001
**Skating distance in fast speed zone (60–79% peak speed)—(m)**			
Defense	984 ± 271	1097 ± 414	0.068
Forwards	1144 ± 343^*^	1326 ± 480^*^	<0.001
**Skating distance in very fast speed zone (80–90% peak speed)—(m)**			
Defense	112 ± 50	131 ± 97	0.158
Forwards	153 ± 66^*^	178 ± 82^*^	0.006
**Skating distance in sprint speed zone (>90% peak speed)—(m)**			
Defense	20 ± 18	23 ± 28	0.461
Forwards	30 ± 24^*^	29 ± 24	0.681

*Data are presented as means ± SD. Values of *p* represent comparisons of means between males and females within the same position*.

* *Represents a significant difference between defense and forwards (*p* < 0.05)*.

### Additional LPS-Derived External Load Measures

Table 4 summarizes average LPS-derived external load measures and comparisons between females and males for defense, forwards, and all players. Males had significantly greater averages in all LPS measures, except for the number of turns and skating transitions in defense. Positional differences between defense and forwards included greater peak acceleration (*p* = 0.022) and deceleration (*p* = 0.002) for male forwards and greater accumulative acceleration load (*p* < 0.001) for female defense. Additionally, female and male defense had more accelerations (*p* < 0.023), decelerations (*p* < 0.001), skating transitions (*p* < 0.001), and direction changes (*p* < 0.001), while female defense had more turns (*p* = 0.006) and male forwards had more impacts (*p* < 0.001).

**Table 4:** Comparison of local positioning system-derived external load between females and males during regular season ice hockey games.

	Females	Males	Value of *p*
**Peak acceleration (m/s^2^)**			
Defense	3.62 ± 0.49	3.98 ± 0.38	<0.001
Forwards	3.70 ± 0.38	4.11 ± 0.45^*^	<0.001
All Players	3.67 ± 0.45	4.06 ± 0.43	<0.001
**Peak deceleration (m/s^2^)**			
Defense	−5.51 ± 0.66	−5.82 ± 0.61	0.005
Forwards	−5.51 ± 0.62	−6.09 ± 0.63^*^	<0.001
All Players	−5.51 ± 0.63	−5.99 ± 0.64	<0.001
**Accumulative acceleration load (AU)**			
Defense	152.1 ± 39.1	189.6 ± 44.4	<0.001
Forwards	131.8 ± 25.9^*^	178.6 ± 57.0	<0.001
All Players	137.9 ± 31.7	182.6 ± 53.1	<0.001
**Number of accelerations**			
Defense	29.2 ± 7.9	48.5 ± 16.5	<0.001
Forwards	25.7 ± 10.5^*^	43.3 ± 16.4^*^	<0.001
All Players	26.7 ± 9.9	45.2 ± 16.6	<0.001
**Number of decelerations**			
Defense	58.7 ± 12.6	65.2 ± 17.0	0.014
Forwards	48.7 ± 13.5^*^	53.7 ± 17.0^*^	0.007
All Players	51.7 ± 14.0	57.8 ± 17.8	<0.001
**Number of turns**			
Defense	59.1 ± 19.0	65.6 ± 27.1	0.115
Forwards	51.7 ± 16.5^*^	66.9 ± 27.6	<0.001
All Players	53.9 ± 17.6	66.5 ± 27.4	<0.001
**Number of skating transitions**			
Defense	82.9 ± 34.3	83.5 ± 30.4	0.982
Forwards	32.9 ± 12.9^*^	48.3 ± 20.3^*^	<0.001
All Players	48.0 ± 31.5	60.6 ± 29.5	<0.001
**Number of direction changes**			
Defense	14.7 ± 5.9	17.7 ± 6.6	0.006
Forwards	10.9 ± 5.0^*^	12.7 ± 5.1^*^	0.003
All Players	12.0 ± 5.6	14.5 ± 6.1	<0.001
**Number of impacts**			
Defense	0.4 ± 0.7	1.9 ± 1.9	<0.001
Forwards	0.9 ± 5.8	3.2 ± 2.7^*^	<0.001
All Players	0.7 ± 4.8	2.7 ± 2.5	<0.001

*Data are presented as means ± SD. Values of *p* represent comparisons of means between males and females within the same position*.

* *Represents a significant difference between defense and forwards (*p* < 0.05)*.

## Discussion

This study used a LPS to quantify the external load of female and male ice hockey players during regular season games. The main findings included that (i) both female and male players had high external load measures during regular season games when compared to other ice hockey research, (ii) in-game LPS-derived measures of external load were greater in males compared to females, (iii) both female and male forwards had greater external load at higher intensities, while defense had greater external load at lower intensities, and (iv) relative speed zones allowed for an adjustment of skating distance intensities for an individualized approach of measuring external load specific to sex and position.

### External Load of Female and Male Ice Hockey Players

Along with physiological sex differences, the differences in gameplay between females and males (e.g., bodychecking is permitted in men’s hockey) allowed for the expectation that external loads would differ between sex. Female players were smaller and younger than male counterparts and most external load measures were different between females and males. Previous research examining heart rate-derived training impulse (TRIMP) as a measure of internal load for the same population reported that male forwards had greater TRIMP than female forwards, while female defense had greater TRIMP than male defense (Bigg et al., 2021). In the current study, all differences suggest that males had a higher external load than females during regular season games. Peak skating speeds for males (defense: 30.15 ± 1.68 km/h; forwards: 31.73 ± 5.0 km/h) and females (defense: 28.52 ± 1.74 km/h; forwards: 28.82 ± 1.39 km/h) were greater than the peak speeds (defense: 24.9 ± 5.0 km/h; forwards: 26.9 ± 5.0 km/h) reported in elite junior U20 Canadian players (Douglas and Kennedy, 2020). Regardless, it appears that external loads need to be considered independently for each sex due to their difference in gameplay.

Similarly, even though males (defense: 5617 ± 1441 m; forwards: 5250 ± 1828 m) skated further than females (defense: 5154 ± 1144 m; forwards: 4206 ± 1037), skating distances for both sexes were greater or comparable than those reported in other studies, which is interesting given the differences in gameplay between sex and skill level. In-game skating distances have been reported as 4606 ± 219 m in national hockey league players (Lignell et al., 2018), 4002.4 ± 786.7 and 3681.2 ± 1058.2 m in elite junior U20 Canadian male defense and forwards (Douglas and Kennedy, 2020), and 5,980 ± 199 m in elite junior Danish U20 male players (Vigh-Larsen et al., 2020). Skating transitions and turns in defense were two measures that did not differ between females and males, outlining the importance of position-specific attention regardless of sex.

One consideration is that both teams in the current study competed on Olympic-size ice, which may have allowed for higher external loads than smaller ice surfaces. Given the difference in anthropometrics, physiology, and sport rules between sexes, different external loads during games were expected (Hunter, 2014). However, it is important to note that even though female’s external load was lower, their skating speeds and distances are still very high when compared with those reported in other studies (Lignell et al., 2018; Douglas and Kennedy, 2020; Vigh-Larsen et al., 2020). This study adds to the limited external load data for ice hockey players and was the first study to report LPS-derived skating speed and distance in games for female players. These data offer insight to the external workload of female players during games and the necessity for specific training requirements to perform optimally during games. Similarly, this shows that decisions and implementation of new strategies should be based on specific athlete demands and workloads when monitoring athlete load.

### External Load of Defense and Forwards

Defense had a greater external load when considering skating distance (only females), accumulative acceleration load and number of accelerations, decelerations, turns (only females), skating transitions, and direction changes. However, male forwards had greater peak speed, acceleration, and deceleration. Similar to the present study, previous research has outlined that defense had greater duration or distance of external load (Lignell et al., 2018), but forwards typically had greater intensities of external load. A study using accelerometry to measure external load for elite female practices and games reported greater PlayerLoad and explosive efforts in forwards when compared to defense (Douglas et al., 2019b). Additionally, a separate study examining the same population reported that explosive ratio and percentage of high force strides were greater in wins compared to losses for forwards only (Douglas et al., 2019a). Further research investigating this relationship using LPS measures may be attractive when considering athlete load and performance outcomes.

The results from the current study show that both female and male forwards have a greater external load at higher intensity skating, while defense have a greater external load at lower intensity skating and in several other LPS metrics (both absolute and relative zones). Studies investigating male players have reported forwards skating at higher intensities during elite junior and National Hockey League (NHL) games (Lignell et al., 2018; Douglas and Kennedy, 2020). While American Hockey League (AHL) defense was shown to train at a greater relative intensity, forwards had a greater intensity of external load during AHL games, with overall load in the game similar between positions (Allard et al., 2020). Based on these results and the consistent positional differences identified, strategies should attempt to develop position-specific training loads to maximize preparation for games, especially if forwards are required to skate at faster speeds during the games as they pressure the defense of the opposing team.

### Skating Distance in Absolute vs. Relative Speed Zones as a Measure of External Load

The most important measure of external load in ice hockey is not yet known, but several measures should likely be considered so that workload and performance can be examined separately. Workload considering skating distance in speed zones of various thresholds includes a reasonable measure of intensity like internal load measures that use heart rate zones as a measure of intensity. As expected, skating distances were different between in absolute and relative speed zones for both sexes, with potential for skating distances at very slow, very fast, and sprint speeds to be overestimated and skating distances at slow and moderate speeds to be underestimated when using absolute speed zones (Figure 2).

While current speed thresholds adapted from other team sports have provided the ability for preliminary investigation of this measure (Lignell et al., 2018), the sex and positional external load differences highlighted above outline the importance of taking an individualized approach. Research in rugby has suggested that this method may account for differences in fitness levels or positional game requirements, optimizing preparation and avoiding under- or overtraining that may affect performance or risk of injury (Clarke et al., 2015; Reardon et al., 2015; Gabbett, 2016; Takamori et al., 2020; Nyman and Spriet, 2021). However, more research is needed to investigate the exact intensity thresholds that should be used, given that physiological biomechanical, and metabolic differences during exercise may all play a role in properly classifying workload measures (Ortega et al., 2021). Regardless, using the peak speed from players during gameplay in the regular season allowed for an individualized measure of skating distance in speed zones and one that is more appropriate for understanding the different in-game demands for individuals of difference sex and position when monitoring external load.

## Conclusion

This was the first study to report LPS-derived external load for female ice hockey players in games and compare this data to male ice hockey players. It was determined that while all players in the study had a high external load during regular season games, the external load was greater in males compared to females. Results showed that forwards have a greater external load at higher intensities and defense has a greater external load at lower intensities. Lastly, given the differences in external load between sexes and positions, relative measures of external load should be considered to provide an individualized approach to athlete load monitoring and specific training to prepare for optimal fitness and performance during games.

## Data Availability Statement

The raw data supporting the conclusions of this article will be made available by the authors, without undue reservation.

## Ethics Statement

The studies involving human participants were reviewed and approved by University of Guelph Research Ethics Board. The patients/participants provided their written and oral informed consent to participate in this study.

## Author Contributions

AG, JB, and LS designed the study. AG, JB, and DN collected on-ice data. AG and JB provided expertise with the local positioning system data collection. AG analyzed all data. AG and LS interpreted it and wrote the manuscript. All authors contributed substantially to revision and approved the final submission.

## Funding

Student support for AG and JB, and funding of equipment was provided by PepsiCo. and Mitacs.

## Conflict of Interest

The authors declare that the research was conducted in the absence of any commercial or financial relationships that could be construed as a potential conflict of interest.

## Publisher’s Note

All claims expressed in this article are solely those of the authors and do not necessarily represent those of their affiliated organizations, or those of the publisher, the editors and the reviewers. Any product that may be evaluated in this article, or claim that may be made by its manufacturer, is not guaranteed or endorsed by the publisher.
